# Understanding functional miRNA–target interactions *in vivo* by site-specific genome engineering

**DOI:** 10.1038/ncomms5640

**Published:** 2014-08-19

**Authors:** Andrew R. Bassett, Ghows Azzam, Lucy Wheatley, Charlotte Tibbit, Timothy Rajakumar, Simon McGowan, Nathan Stanger, Philip Andrew Ewels, Stephen Taylor, Chris P. Ponting, Ji-Long Liu, Tatjana Sauka-Spengler, Tudor A. Fulga

**Affiliations:** 1 Medical Research Council Functional Genomics Unit, Department of Physiology, Anatomy and Genetics, University of Oxford, South Parks Road, Oxford OX1 3PT, UK; 2 Radcliffe Department of Medicine, Weatherall Institute of Molecular Medicine, University of Oxford, Oxford OX3 9DS, UK; 3 Computational Biology Research Group, Radcliffe Department of Medicine, Weatherall Institute of Molecular Medicine, University of Oxford, Oxford OX3 9DS, UK; 4 Science for Life Laboratory, Department of Biochemistry and Biophysics, Stockholm University, Stockholm SE-106 91, Sweden; 5 These authors contributed equally to this work; 6 Present address: Dunn School of Pathology, University of Oxford, South Parks Road, Oxford OX1 3RE, UK; 7 Present address:School of Biological Sciences, Universiti Sains Malaysia, 11800 Penang, Malaysia

## Abstract

MicroRNA (miRNA) target recognition is largely dictated by short ‘seed’ sequences, and single miRNAs therefore have the potential to regulate a large number of genes. Understanding the contribution of specific miRNA–target interactions to the regulation of biological processes *in vivo* remains challenging. Here we use transcription activator-like effector nuclease (TALEN) and clustered regularly interspaced short palindromic repeat (CRISPR)/Cas9 technologies to interrogate the functional relevance of predicted miRNA response elements (MREs) to post-transcriptional silencing in zebrafish and *Drosophila*. We also demonstrate an effective strategy that uses CRISPR-mediated homology-directed repair with short oligonucleotide donors for the assessment of MRE activity in human cells. These methods facilitate analysis of the direct phenotypic consequences resulting from blocking specific miRNA–MRE interactions at any point during development.

## Abstract (web summary)

Identifying miRNA response elements (MREs) within target mRNAs can be done computationally but the functional validation of putative MREs remains challenging. Here, Bassett *et al*. describe applications of genome engineering to target and assess the functional significance of MREs in different organisms and stages of development.

## Graphical abstract

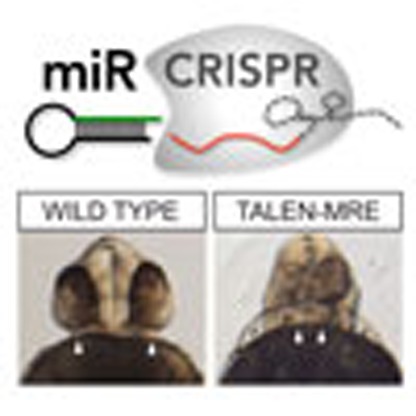

MicroRNA (miRNA)-mediated post-transcriptional silencing represents an essential regulatory layer underlying cellular function, development and disease^1^,^2^,^3^. Because the specificity for miRNA target recognition is largely dictated by short ‘seed’ sequences (nucleotides 2–8 in mature miRNAs), miRNAs can potentially regulate large numbers of genes^4^. Although advances in bioinformatics algorithms have increased the confidence of miRNA target predictions, the false-positive rate of such *in silico* approaches remains very high (~60%)^5^. In response, genome-wide experimental approaches have been developed to map physical interactions between miRNAs and target mRNAs^6^,^7^, and yet these are not suitable for assessing the functional relevance of such interactions in the context of a living cell. Understanding the significance of miRNA response elements (MREs) *in vivo* has thus remained challenging, and the biological function of only a vanishingly small proportion of these interactions has been established experimentally. To date, the standard strategy used to validate miRNA targets has relied on artificial sensor assays whereby the 3′-untranslated region (UTR) of the gene of interest (or a region flanking the predicted MRE) is coupled to a reporter gene. Although valuable, a critical limitation of this technology is its inability to infer the physiological relevance of a putative MRE *in vivo,* as these assays do not recapitulate the endogenous context and stoichiometry of miRNAs and their targets. An alternative strategy is provided by target protector (TP) oligonucleotides, which overlap with the seed region and a unique flanking sequence in the target 3′UTR, and can therefore block miRNA access to the MRE^8^,^9^,^10^,^11^. Although suited for validation of MREs *in vivo*, few studies have been performed to address potential toxicity or off-target effects of TP oligonucleotides. Furthermore, TP assays are in most cases limited to transient effects in systems where oligonucleotides can be supplied by injection or transfection^12^.

To address these limitations, we have used genome engineering technologies to genetically ablate endogenous MREs within putative target genes *in vivo*. To generate targeted insertion and deletion (indel) mutations within MREs, we use both transcription activator-like effector nucleases (TALENs) in zebrafish and the clustered regularly interspaced short palindromic repeats (CRISPR)-associated nuclease (Cas9) in *Drosophila*. In both systems, indels are created as a result of inefficient non-homologous end joining repair at the nuclease cleavage site. Using this approach, we have accurately defined *bona fide* MREs in these two species and have directly addressed their functional relevance in an endogenous context *in vivo*. Furthermore, we describe a novel approach for the identification of active MREs in cell culture systems, using CRISPR-mediated homology-directed repair (HDR) with short oligonucleotide donors. This now allows the effect of defined deletions or alterations within MREs to be rapidly assessed in heterogeneous cell populations. Finally, we present an online algorithm that computationally predicts CRISPR target sites neighbouring all predicted MREs in several species, and thereby show that these techniques may be generally applicable for investigation of the vast majority of MREs.

## Results

### Generating targeted MRE deletions in zebrafish using TALENs

**Figure 1:**
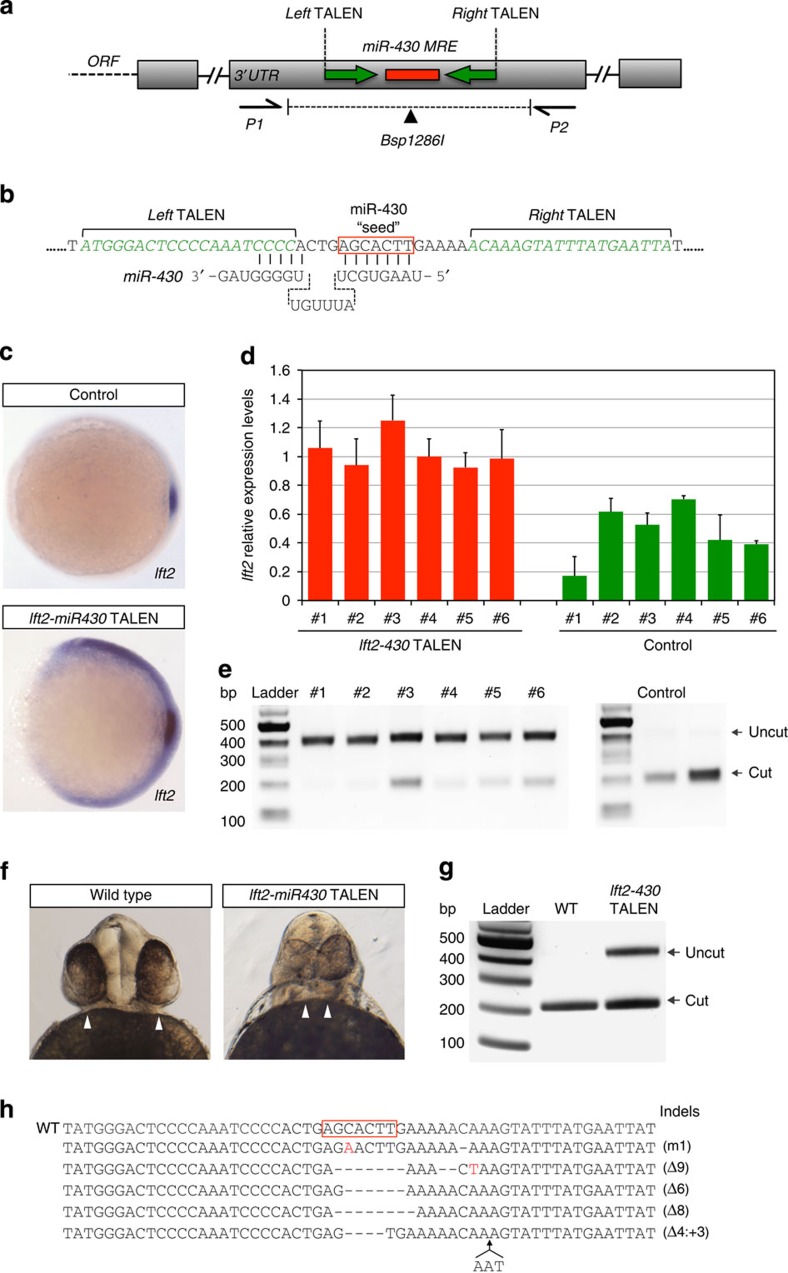
miR430-mediated regulation of *lft2* expression in zebrafish. (**a**) Schematic diagram of the *lft2* 3′UTR showing positions of TALEN-binding sites (green arrows), miR-430-binding site (MRE, red box), primers used to amplify the region (P1, P2) and the *Bsp1286I* restriction site (black triangle). (**b**) Detail of miR-430 MRE genomic region showing the position of TALEN-binding sites (green) and the miR-430 seed sequence (red box). (**c**,**d**) *lft2*-miR430 TALEN-injected animals show increased *lft2* expression at shield stage, as detected by *in situ* hybridization (**c**), or qPCR analysis of single embryos (**d**) (error bars=s.e.m. of three technical replicates). (**e**) Validation of miR-430 MRE disruption by loss of the *Bsp*1286I restriction site. Gel image shows PCR products of genomic DNA isolated from embryos in **d** digested with *Bsp1286I*. (**f**) Cycloptic phenotype in *lft2*-miR430 TALEN-injected embryos at 50 h.p.f. (16.4% phenotypic penetrance (*n*=85)). (**g**) Loss of the *Bsp*1286I restriction site in genomic DNA isolated from TALEN-injected embryos in **f**, compared with controls. (**h**) Sequencing of region spanning the TALEN cut site from embryo in **f** shows discrete indels across the predicted miR-430 MRE. Seed sequence is marked by a red box, and deletions, insertions and substitutions are indicated relative to the WT sequence (first line).

In zebrafish, morpholino TP oligonucleotides have been used to block a predicted miR-430 site in the *lefty2* (*lft2)* 3′UTR, causing a midline development phenotype in the embryo as a consequence of impaired Nodal signalling^8^. To provide proof of concept for our approach, we first designed a TALEN pair to genetically ablate this miR-430 site in *lft2* DNA (Fig. 1a,b). Customized TALE repeats fused to a FokI nuclease were assembled using the Golden Gate method^13^, and engineered to target 20 bp and 18 bp flanking the miR-430 target site, with a 16-bp spacer (Fig. 1a,b). The spacer region includes a *Bsp*1286I restriction enzyme-recognition sequence, which overlaps the predicted TALEN cut site, and the seed region of the miR-430 MRE. mRNAs encoding the TALEN pair were injected into single-cell zebrafish embryos to generate chimeric mutant animals^14^. Analysis by *in situ* hybridization and quantitative PCR (qPCR) of single experimental embryos showed that *lft2* expression level at shield stage was significantly increased as compared with controls (*n*=6, average *lft2* increase significance *P*<0.005, Fig. 1c,d), suggesting de-repression of miR-430-mediated regulation. Disruption of the *lft2* miR-430 MRE genomic locus was confirmed in TALEN-injected embryos, by the loss of *Bsp*1286I restriction site (Fig. 1e). In agreement with previous studies^8^, embryos at 56 h.p.f. (hours post fertilization) displayed cyclopia and head defects (Fig. 1f), with a mean phenotypic penetrance of 16.4% (*n*=85). miR-430 MRE disruption, as assayed by the resistance to *Bsp*1286I digest, was observed in DNA samples obtained from all tested embryos that exhibited mutant phenotypes (*n*=8, representative example shown in Fig. 1g). Finally, sequence analysis of the target region demonstrated discrete indel mutations across the predicted miR-430 MRE (Fig. 1h). Overall, the frequency of animals carrying indels at 56 h.p.f. was 96% (*n*=32), indicating highly efficient editing events at the targeted locus. These results establish the feasibility of this approach for analysis of miRNA–target regulatory interactions during zebrafish embryonic development, and validate the functional relevance of this MRE in the *lft2* 3′UTR. Notably, such mutants could be used to generate stable lines, allowing phenotypic analysis at all stages of development and during adult life.

### Generation of MRE deletions in *Drosophila* by CRISPR-Cas9

To realize the potential of our strategy in elucidating the functional significance of MREs throughout development, we next investigated the effect of removing a well-studied target site for the miRNA *bantam* (*ban*) in the *enabled* (*ena*) gene in *Drosophila*. Previous work showed that overexpression of *ban* in the wing imaginal disc can reduce Ena levels in the context of a 3′UTR reporter assay, and that this effect was dependent on a conserved *ban* target site^15^. However, this strategy entails constitutive misexpression of the reporter and overexpression of the miRNA, which do not necessarily reflect the endogenous context and relative amounts of the miRNA and its target.

**Figure 2:**
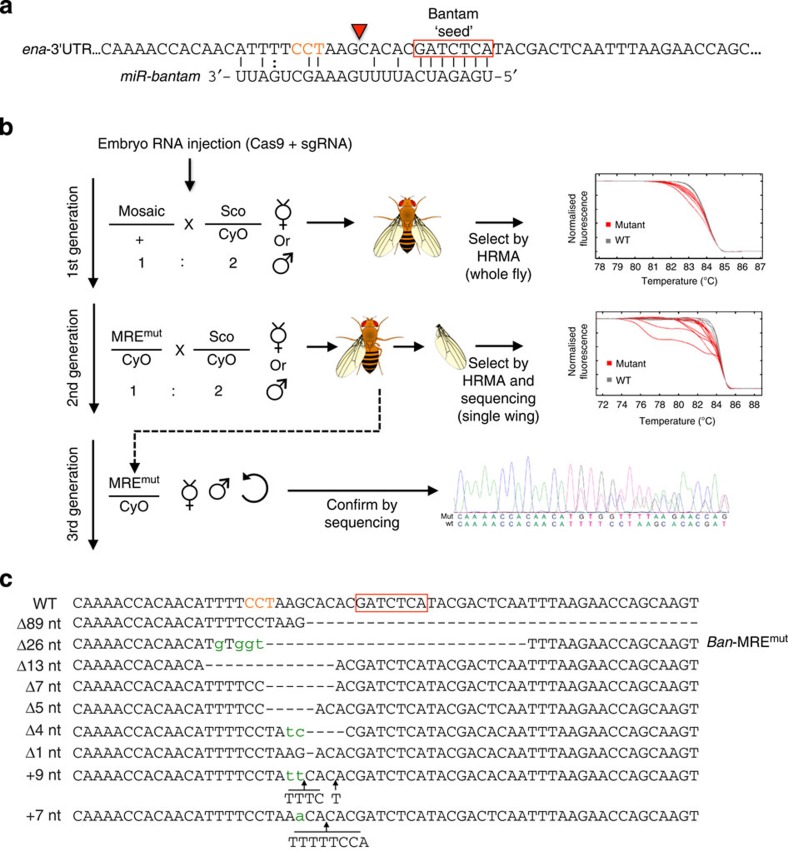
Generation of a *ban* MRE deletion in the *Ena* gene in *Drosophila*. (**a**) Schematic diagram of the *ena* 3′UTR around the *ban* miRNA response element (MRE). The *ban* seed sequence is indicated by a red box, the PAM in orange and the Cas9 cleavage site by a red triangle. (**b**) Generation of *ban* MRE deletion. Potentially mosaic offspring from the CRISPR-injected G_0_ generation are individually crossed to a marker/balancer line (Sco/CyO, first generation). After 1 week, injected flies are removed, DNA is extracted from the whole fly and HRMA performed to identify mosaic animals (upper right panel). Offspring from crosses with confirmed mosaic mutations are isolated, and screened for indel germline transmission by HRMA analysis of DNA isolated from one wing (middle right panel). The nature of potential mutations is established by sequencing, before setting up individual crosses to a balancer line (Sco/CyO, 2nd generation). In the third generation, heterozygous male and virgin female flies are used to establish stable lines and mutations are confirmed by sequencing (lower right panel). (**c**) Nature of the indel mutations generated at the *ban* MRE. An alignment of sequences from wild-type (first line) and mutant lines is shown. The predicted *ban* seed sequence within the *ena* 3′UTR is indicated by a red box, insertions are marked in green or with arrows, and deletions with dashes. The protospacer adjacent motif (PAM) necessary for Cas9 cleavage is indicated in orange type. The *ban*-MRE^mut^ deletion that was used for subsequent analysis is indicated.

**Figure 3:**
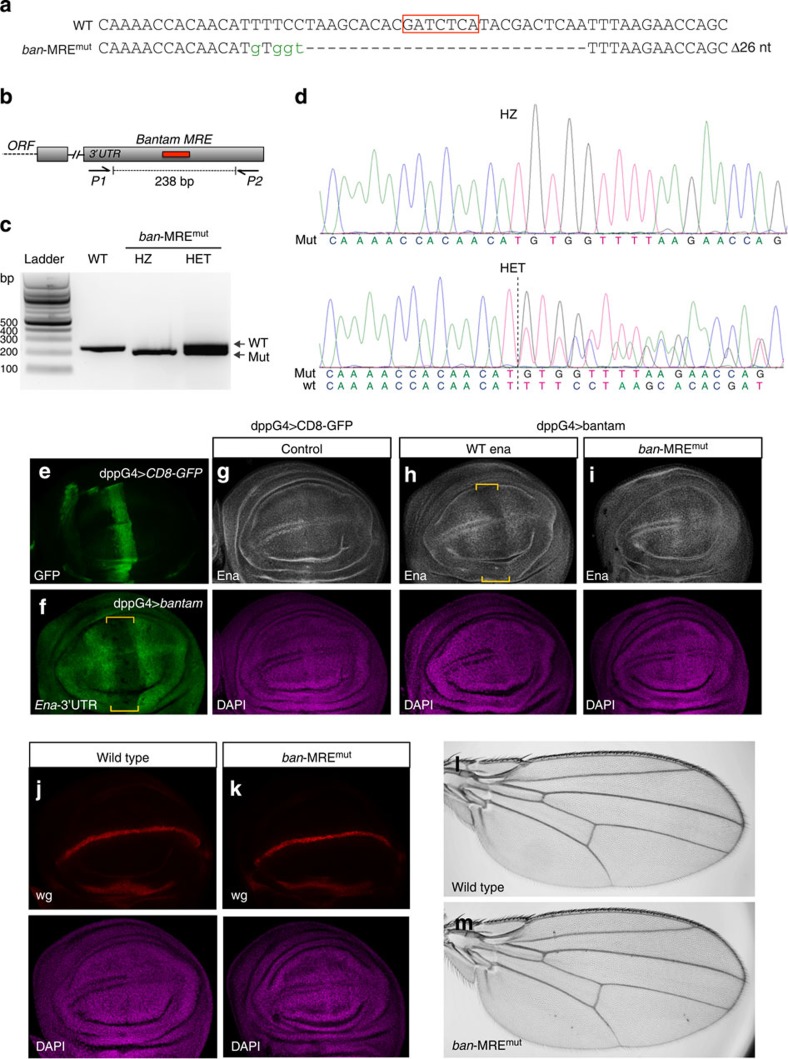
*ban-*mediated regulation of *Ena* expression in *Drosophila*. (**a**) Nature of the *ban*-MRE^mut^ allele of *ena*. Deletions (dashes) and insertions (green lower case) are indicated relative to WT sequence (first line). (**b**,**c**) Primers used to amplify the region around the *ban* target site (**b**), and results of PCR in wild type (WT), homozygous (HZ) and heterozygous (HET) mutants are shown (**c**). (**d**) Results from sequencing of PCR products in homozygous (HZ) and heterozygous (HET) mutants show the nature of the mutation. (**e**–**i**) Analysis of third instar larval wing imaginal discs. (**e**) Expression pattern of *dpp*-GAL4 as revealed by UAS-CD8-EGFP (green). (**f**–**i**) Effect of *ban*-MRE^mut^ on Ena expression (*n*>30). (**f**) Overexpression of *ban* using *dpp*-GAL4 lowers the levels of ubiquitously expressed GFP *ena*-3′UTR-sensor (*ena-*3′UTR). (**g**) Overexpression of CD8-GFP with *dpp*-GAL4 has no effect on Ena protein expression (white). (**h**) Overexpression of *ban* with *dpp*-GAL4 in WT flies reduces endogenous Ena levels (yellow brackets). (**i**) Overexpression of *ban* with *dpp*-GAL4 in homozygous *ban*-MRE^mut^ flies has no effect on endogenous Ena expression. (**j**–**m**) Analysis of wing patterning in homozygous *ban*-MRE^mut^ flies (*n*=10). (**j**) Wild-type discs stained with an anti-wingless (Wg) antibody highlight its expression across the D/V boundary of the disc. (**k**) Homozygous *ban*-MRE^mut^ discs show an identical pattern of wg expression. (**l**,**m**) Adult wings from (**l**) wild-type or (**m**) homozygous *ban*-MRE^mut^ animals show no obvious defects in wing patterning.

To create a genomic deletion in the predicted MRE, we designed a synthetic guide RNA (sgRNA) that would target the Cas9 nuclease to the predicted *ban* target site in the *ena* 3′UTR (Fig. 2a). The experimental workflow underlying generation of stable MRE mutants in *Drosophila* is detailed in Fig. 2b. Briefly, *in vitro* transcribed RNA for Cas9 and the sgRNA were injected into syncytial blastoderm stage *Drosophila* embryos^16^, and chimeric mutant flies were identified by high-resolution melt analysis (HRMA). Mutations transmitted through the germline were followed and validated by HRMA and sequencing of wings from individual offspring. Animals carrying indel mutations across the *ban* target site were then selected, and stable transgenic lines were generated. The identity of each mutation was further confirmed by Sanger sequencing (Fig. 2c). We decided to study the effect on Ena expression of one of these mutants that deleted the entire *ban* target site including the seed sequence (Fig. 3a, *ban-*MRE^mut^). The presence of the mutation in animals used for all subsequent steps was validated by PCR (Fig. 3b,c) and sequencing (Fig. 3d). We overexpressed *ban* using the *decapentaplegic* driver (*dpp*-GAL4) that expresses in a stripe of cells across the anterior/posterior boundary of the wing imaginal disc (Fig. 3e). Confirming previous observations^15^, this resulted in a specific downregulation of a GFP *ena*-3′UTR-sensor construct (Fig. 3f, *ena-*3′UTR) and endogenous Ena protein levels (Fig. 3h, *n*>30). No change in Ena levels was observed in control discs expressing CD8-EGFP under the same GAL4 driver (Fig. 3g). When the same experiment was performed in homozygous mutant *ban*-MRE^mut^ flies, no reduction was observed in the levels of Ena protein (Fig. 3i, *n*>30). This result indicates that overexpressed *ban* can directly target *ena* via this predicted MRE in its 3′UTR.

It was previously suggested that endogenous *ban* regulates Ena expression on either side of the dorsal/ventral (D/V) boundary of the wing imaginal discs^15^. Nevertheless, in contrast to expectations, analysis of the homozygous *ban*-MRE^mut^ animals revealed no apparent changes to endogenous Ena expression pattern in the wing imaginal discs (compare Fig. 3g and Fig. 3i). We also did not observe any obvious defects in the D/V boundary as revealed by wingless staining (Fig. 3j,k, *n*=10), or any detectable phenotypes in the adult wings (Fig. 3l,m, *n*=10). This indicates that although *ban* overexpression is able to downregulate Ena through this MRE, the endogenous expression pattern of Ena in the wing disc is not dependent on this target site. Thus, although it remains possible that *ban* participates in establishing the identity of the D/V boundary in wing discs, it is unlikely this activity is mediated by tuning Ena expression through this predicted MRE. These results emphasize the importance of directly establishing the *in vivo* physiological relevance of a MRE, and suggest that caution should be exerted when inferring its function from indirect assays.

### Assessing MRE activity in human cells by CRISPR-mediated HDR

**Figure 4:**
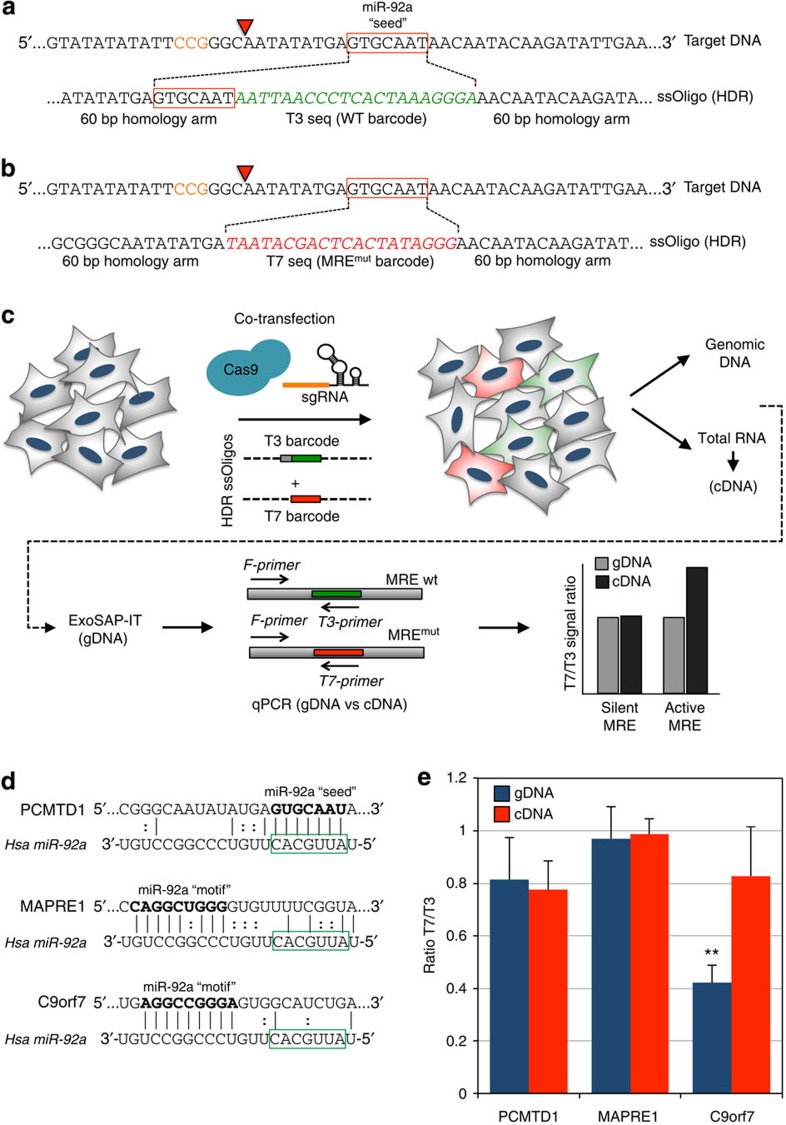
Analysis of MRE activity in human cells by CRISPR-mediated HDR. (**a**,**b**) Design of homology oligonucleotides for CRISPR-enhanced homology-directed repair (HDR). The PAM is indicated in orange, and cleavage site by a red triangle. (**a**) The T3 oligonucleotide contains 60 nt homology arms flanking the miR-92a MRE (seed sequence indicated by a red box), to introduce a T3 primer-binding site (green italic, WT barcode) and maintain the miR-92a MRE. (**b**) The T7 oligonucleotide contains similar 60 nt homology arms, but deletes the miR-92a seed sequence, and introduces a T7 primer-binding site (red italic, MRE^mut^ barcode). (**c**) Outline of experimental design. A plasmid expressing Cas9 and the sgRNA targeting the MRE is co-transfected into HEK293T cells along with the two HDR donor oligonucleotides. This results in a population of cells with a proportion containing the T3 oligonucleotide integrated at the MRE (green) and a proportion containing the T7 oligonucleotide integrated at the MRE site, but deleting the MRE (red). Genomic DNA (gDNA) and RNA are extracted, and the gDNA treated with ExoSAP-IT to remove any contaminating donor oligonucleotides. gDNA and cDNA are subjected to qPCR analysis using a gene-specific forward primer (F-primer) and T3- or T7-specific reverse primers (green and red). The ratio of T7 signal to T3 signal in the gDNA is used to estimate the relative integration efficiency of the T7 and T3 oligos. Any increase in this ratio in the cDNA suggests that the MRE is active, as its deletion results in increasing amounts of transcript being produced. (**d**) Target genes tested in this system. Three MREs identified by CLASH analysis, and whose expression appears upregulated upon loss of miR-92a function were analysed^7^. PCMTD1 contains a classical ‘seed’ sequence, whereas MAPRE1 and C9orf7 contain non-canonical binding motifs. Complementary base pairing is indicated, and motifs deleted by HDR are highlighted in bold (miR-92a ‘seed’ or miR-92a ‘motif’). The seed region of miR-92a is highlighted by a green box. (**e**) Analysis of MRE functionality in PCMTD1, MAPRE1 and C9orf7. The ratio of T7 signal to T3 signal is displayed on the *y* axis, and results from gDNA are shown in blue and cDNA in red. Only C9orf7 appears to be upregulated upon MRE deletion (*P*=0.0082, one-tailed *T*-test, *n*=3 independent transfections). Error bars show 95% confidence intervals of three biological replicates.

Although studies *in vivo* are critical for understanding the function of MREs in the context of the whole organism, cell culture systems offer a valuable tool for studying miRNA function in a well-defined context, and a means to directly investigate their role in human cells. We therefore developed a novel strategy to validate functional MREs in cultured cells, using the CRISPR/Cas9 system to enhance homologous recombination, and generate defined sequence alterations of a MRE of interest. The approach involves co-transfection of the Cas9/sgRNA targeting a MRE with two ~140 nt single-stranded DNA (ssDNA) oligonucleotide templates for HDR. One of these HDR templates inserts a T3 ‘barcode’ downstream of the target MRE locus, but maintains the intact MRE (Fig. 4a), whereas the second deletes the MRE and replaces it with a T7 ‘barcode’ (Fig. 4b). The two populations of cells can therefore be distinguished in a pooled sample using primers binding the T3 or T7 ‘barcodes’ and a common primer specific for the MRE of interest (Fig. 4c). This allows the activity of the MRE to be assessed in a heterogeneous mixture of transiently transfected cells. In the case of a functionally active MRE, higher levels of the mRNA containing the T7 ‘barcode’ where the MRE has been deleted are expected, compared with the T3 ‘barcode’, where the MRE remains intact. This can be quantified by qPCR with the T7 and T3 primers on complementary DNA (cDNA) from the pool of cells. The integration efficiencies of the T7 and T3 oligonucleotides are assessed by qPCR of genomic DNA (gDNA) isolated from the same pool of cells, and compared to the results obtained with the cDNA sample. When the ratio of T7/T3 signal is significantly higher in the cDNA than the gDNA, then the MRE is active in this cell type (Fig. 4c), and its increase indicates the magnitude of the effect.

We tested this experimental strategy in HEK293T cells with three putative targets of miR-92a that were identified by Crosslinking, Ligation And Sequencing of Hybrids (CLASH)^7^. Although, in general, CLASH binding results were supported by transcript upregulation upon miR-92a inhibition^7^, this assay cannot discriminate between direct and indirect miRNA-mediated regulation. One such target we tested (PCMTD1) carried a classical ‘seed’ sequence within its MRE, whereas two others (MAPRE1, C9orf7) contained non-canonical binding sites, defined by a ‘motif’ complementary to the 3′ end of the miRNA (Fig. 4d). Our analysis revealed that despite predicted direct miRNA–MRE binding, in only one case (C9orf7) did deletion of the putative MRE confer an effect on transcript abundance (Fig. 4e, *n*=3). Interestingly, C9orf7 is targeted by miR-92a via a non-canonical motif through complementary base pairing with its 3′ end, demonstrating that such interactions can be biologically relevant. The other putative MREs in PCMTD1 and MAPRE1 failed to show this effect, suggesting that they may not play a role in regulating the abundance of these transcripts, at least in this particular cell type. One consideration when designing this strategy is to determine whether integration of the barcodes creates or inadvertently removes endogenous MREs. To test this, the sequences generated upon integration of T7-MRE^mut^ and T3-MRE^WT^ barcodes were screened for miRNA target sites using the PITA algorithm^17^. This analysis revealed that no endogenous MREs were removed, except for the miR-92 family following integration of the T7-MRE^mut^ barcode, as intended (Supplementary Table 1). All but one novel MREs were intrinsic to the T3 or T7 sequences and therefore consistent across all target genes. The miR-944 MRE introduced by the T3-MRE^WT^ integration in PCMTD1 was considered inconsequential, as miR-944 is not present to any detectable levels in HEK-293 cells^18^. We also performed analogous experiments in *Drosophila* S2R+ cells with two predicted targets of miR-184 (PCK and CG13088), and revealed that both of these MREs are functional in this cell type (Supplementary Fig. 1).

Based on these results, we propose that this strategy can be applied broadly to the identification of physiologically active MREs when the target mRNA is expressed at sufficient levels to allow detection by qPCR of homologous integration within the cDNA. In addition, our results suggest that care must be taken when extrapolating the functional relevance of a MRE from studies of miRNA binding to the target transcript or changes in gene expression resulting from miRNA inhibition.

### miR-CRISPR: a genome-wide CRISPR design resource for MREs

**Figure 5:**
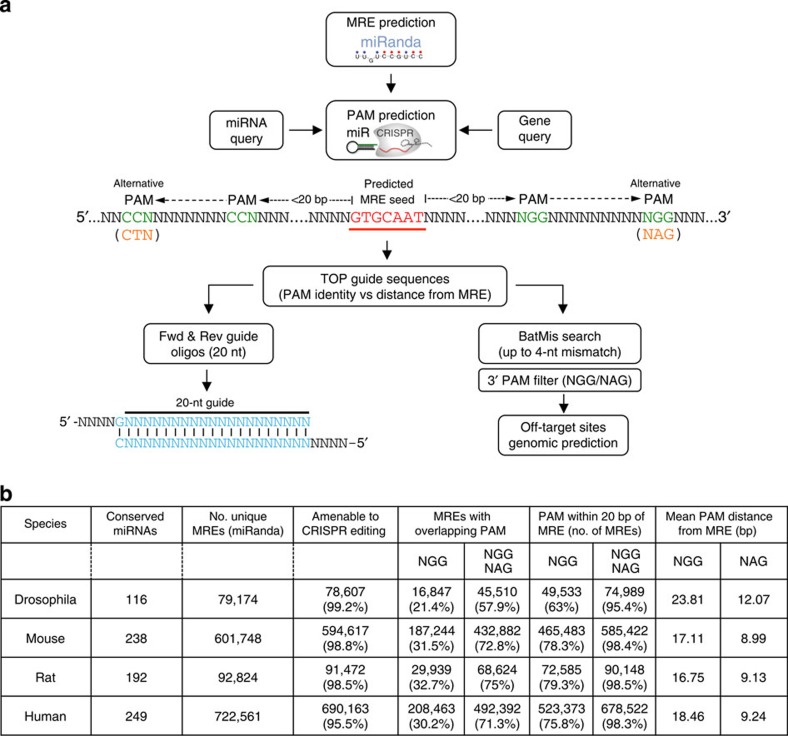
*In silico* prediction and design of CRISPR sgRNAs targeting putative MREs in four species. (**a**) Overview of computational strategy used by the miR-CRISPR algorithm to define MREs amenable to CRISPR editing. MREs for all conserved miRNAs were predicted in all genes in *Drosophila*, mouse, rat and human using the miRanda algorithm. The closest PAM sequences to the MRE seed sequence are output, along with the CRISPR guide RNA sequences necessary to target them. For each guide sequence, off-targets are identified by searching for sequences within the genome containing up to four mismatches to the guide sequence. (**b**) MREs amenable to CRISPR editing in *Drosophila*, mouse, rat and human. Columns in the table show the number of conserved miRNAs present, the number of unique MREs predicted by the miRanda algorithm and the number and percentage of these MREs that are amenable to CRISPR editing. CRISPR target sites are divided in two categories based on their distance from the MRE. Metrics are provided for those where the PAM overlaps with the seed sequence in the MRE, or those where the PAM is within 20 nt of the MRE. These are further categorized depending on whether they contain a NGG PAM sequence or whether both NAG and NGG sequences are considered. The mean distance from the MRE is also indicated for each PAM class.

To establish the versatility of these technologies, and whether they can be generally applied to analysis of miRNA–MRE interactions, we performed computational analysis to identify CRISPR target sites in the vicinity of all predicted MREs in several organisms (Fig. 5a). CRISPR target sites are 20 nt long and require a downstream protospacer adjacent motif (PAM), which can take the form of NGG or NAG. Efficiency of cleavage is higher with the NGG sequences, and therefore this PAM is preferred if possible. MREs for all conserved miRNAs and predicted by the miRanda algorithm with a ‘good mirSVR score’ (http:// www.microrna.org, August 2010 release^19^,^20^) were included in this analysis. sgRNA target sequences were identified in the proximity of these sites from the presence of NGG or NAG sequences on either strand, flanking the MRE of interest. These were divided into two classes, those where the PAM sequence overlapped with the MRE, and those where it lay within 20 bp of the MRE (Fig. 5a). This analysis revealed that across all species analysed, between 95.4 and 98.5% of putative MRE genomic loci carry NGG or NAG PAMs either inside or within 20 bp of the MRE, making them amenable to CRISPR-mediated genome engineering (Fig. 5b). Coupled with the ability to generate large libraries of sgRNAs^21^,^22^, this suggests that the analyses described here can be extended to larger numbers of MREs.

CRISPR target sites for all predicted MREs are available via the miR-CRISPR online web interface ( http://miR-CRISPR.molbiol.ox.ac.uk/fulga/miR-CRISPR.cgi). Information is provided for five species: human, mouse, rat, *Drosophila* and *Caenorhabditis elegans*. Upon entering a gene symbol and/or miRNA identifier, CRISPR sgRNA target sites are generated for each predicted MRE, along with the distance between the PAM and MRE, the forward and reverse oligonucleotides required for sgRNA production, and the number and details of putative off-target sites.

## Discussion

Understanding the cellular networks regulated by miRNAs requires identification of their direct endogenous targets *in vivo*. This knowledge is also essential when anticipating the broad consequences of manipulating miRNA function for therapeutic intervention. Here we used genome engineering technologies to genetically alter miRNA target sites within the 3′UTRs of several genes, and explored their functionality *in vivo*. Because most MREs are not within open reading frames and are relatively short, small non-homologous end joining-based deletions are ideally suited for their manipulation, and allow investigation of miRNA target genes in the context of any organism amenable to genome engineering. We also describe a novel system for the study of MREs in cell culture systems that uses HDR enhanced by CRISPR nucleases to make defined changes to MREs, and analyze the resulting effects on transcript abundance. These results demonstrate that not all putative MREs are functional in their endogenous context. Furthermore, they suggest that although 3′UTR reporter systems, miRNA gain- or loss-of-function, or direct miRNA-target binding assays are valuable experimental tools, they are not always sufficient to reliably predict MRE activity *in vivo*. This highlights the importance of defining the physiological activity of MREs in the context of a living system. The unique experimental framework described here can be applied to the majority of predicted MREs in many experimental systems, and requires relatively minor time and resource inputs, comparable with TP and 3′UTR reporter assays. In addition, it enables a definitive interrogation of physiological miRNA–target interactions in their endogenous context and thus permits their functional relevance to be established *in vivo*.

## Methods

### TALEN design and assembly

The TALE nuclease was designed using the TALEN Targeter algorithm ( https://tale-nt.cac.cornell.edu/node/add/talen)^23^. Repeat variable di-residue (RVD) arrays were designed to target 20 and 18 nt flanking the *lft2* 3′UTR at the miR-430-binding site, separated by a 16-nt spacer. RVD arrays containing HD, NG, NI and NN monomers were assembled using the Golden Gate TALEN and TAL Effector Kit 2.0 (Addgene, #1000000024) as previously described^13^. The final TALENs pairs were cloned into a pMTB2-Goldy vector generated by inserting the Goldy TALEN ORF into a Tol2-based integration vector under the control of the β-actin2 promoter^14^. This construct allows stable integration into the zebrafish genome, as well as *in vitro* transcription of corresponding TALEN mRNAs. The RVD sequences of the left and right TALENs are:

*Lfty2* TALEN A: NI NG NN NN NN NI HD NG HD HD HD HD NI NI NI NG HD HD HD HD

*Lfty2* TALEN B: NI NI NG NG HD NI NG NI NI NI NG NI HD NG NG NG NN NG.

### Production of TALEN mRNA

Plasmid pMTB2-Goldy *Lfty2* TALEN A and *Lfty2* TALEN B constructs were linearized with *Pme*I (NEB, #R0560), and capped TALEN mRNAs were *in vitro* transcribed using the mMESSAGEmMACHINE SP6 kit (Ambion, #AM1340). RNAs were then polyadenylated with the polyA tailing kit (Ambion, #AM1350) and purified with the RNeasy mini kit (Qiagen, #74104).

### Zebrafish injections

A measure of 25 pg of each TALEN mRNA was injected into single-cell stage embryos, using the Picospritzer II microinjector (Parker Instrumentation). Embryos were incubated in E3 medium^24^ at 28.5 °C, and either fixed with 4% PFA at shield stage or 56 h.p.f. or lysed for transcript/gDNA analysis. For phenotypic assessment, three independent rounds of injection were performed, and a minimum of 85 surviving embryos were analysed from each round. The mean penetrance of the observed phenotype was 16.4% (s.d.=3.2). Injections were performed in wild-type zebrafish AB or TU strains, which were used interchangeably. All experiments were performed in wild-type embryos up to 5 days post fertilization, and as such were not subject to Home Office Regulations.

### *In situ* hybridization analysis of zebrafish embryos

The template for *lft2* antisense probe was generated by PCR amplification of cDNA from 12 hours post fertilization wild-type zebrafish, using the following primers: SP6-lft2-F 5′-GATTTAGGTGACACTATAGgaccacagcgatctcactca-3′ and T7-lft2-R 5′-TAATACGACTCACTATAGGGgactggagggattttgtcc-3′. The PCR product was gel extracted and purified by ethanol precipitation. 1.5 μg of template DNA was transcribed using T7 RNA polymerase (Promega, #P207B) in the presence of digoxygenin (DIG)-labelled dNTPs (Roche, #12430721). Probes were purified using G-50 micro-columns (GE Healthcare, #28-9034-08). Embryos were permeabilized with 1% H_2_O_2_, equilibrated with hybridization buffer (50% Formamide, 1.3 × SSC pH 5.0, 5 mM EDTA pH 8.0, 200 μg ml^−1^ Baker’s yeast tRNA, 0.2% Tween-20, 0.5% 3-[(3-cholamidopropyl)dimethylammonio]-1-propanesulfonate (CHAPS), 100 μg ml^−1^ Heparin) and incubated overnight with *lft2* DIG-labelled probes. Embryos were blocked with 20% sheep serum+2% Boehringer Blocking Reagent (Roche, #11096176001). Probes were detected with an anti-DIG-alkaline phosphatase antibody (Roche, #11093274910) and the signal was developed using 30 μg ml^−1^ of each nitro-blue tetrazolium chloride and 5-bromo-4-chloro-3-indolyl-phosphate (Roche, #11383213001/#11383221001).

### Restriction enzyme mapping and sequencing

gDNA was extracted by homogenizing single zebrafish embryos in 50 μl of 50 mM NaOH, followed by incubation for 20 min at 95 °C, cooling to 4 °C and addition of 5 μl of 1 mM Tris-HCl pH=8 to neutralize the solution^25^. A 408-bp fragment spanning the miR-430 target site was amplified from wild-type or TALEN-injected zebrafish gDNA using Phusion DNA polymerase (NEB, #M0532) and flanking primers lfty2-Fwd (5′-CCCATGATGTACCTGGTCAAAA-3′) and lfty2-Rev (5′-GCTGTGGTGACCCCTAATGAAT-3′). The PCR product was then purified using the QIAquick Gel Extraction Kit (Qiagen, #28706) and digested with *Bsp*1286I (NEB, #R0120) for 4 h at 37 °C. Digested products were then analysed by gel electrophoresis (1% agarose). To identify the nature of indel mutations, PCR products were cloned into pGEM-T easy vector (Promega, #A1360) and DNA was isolated from individual clones and sequenced using an SP6 primer.

### Expression analysis of zebrafish embryos

For coupled transcriptional/genomic analysis of single injected zebrafish embryos, total RNA and DNA were extracted using the RNAqueous Micro Kit (Ambion, #AM1931). cDNA was synthesized using the Superscript III Reverse transcriptase kit (Invitrogen, #18080-044). For quantitative PCR, the following gene-specific primers were used: Lft2-F (5′-CTGGCAGGAATACTCAGGGG-3′), Lft2-R (5′-TGGCCTCCATGTCGAACA-3′), ActB-F (5′-AATCCCAAAGCCAACAGAGA-3′), ActB-R (5′-ACATACATGGCAGGGGTGTT-3′). The levels of Lft2 transcript in individual embryo analysis were calculated using the standard curve method.

### CRISPR design and production for *Drosophila*

The target sequence was chosen on the antisense strand of the DNA nearest to the miRNA-binding site (N_20_NGG). Potential off target sites within the *Drosophila* genome were identified by BLAST and using the CRISPR design tool ( http://crispr.mit.edu). The closest off-target site contained four mismatches to the 20 nt target sequence. Cas9 mRNA was *in vitro* transcribed from plasmid MLM3613 (ref. 26; Addgene, #42251) using the mMESSAGE mMACHINE T7 kit (Ambion, #AM1344) as described above. sgRNA template DNA was generated by PCR with Phusion polymerase (New England Biolabs) in HF buffer with a unique oligonucleotide encoding the T7 polymerase-binding site (underlined) and the sgRNA target sequence (italic; enaRNA-F=5′-GAAATTAATACGACTCACTATA*GGCGTATGAGATCGTGTGCTT*GTTTTAGAGCTAGAAATAGC-3′) and a reverse oligonucleotide encoding the remainder of the sgRNA sequence (sgRNA-R=5′-AAAAGCACCGACTCGGTGCCACTTTTTCAAGTTGATAACGGACTAGCCTTATTTTAACTTGCTATTTCTAGCTCTAAAAC-3′). Reaction solutions (100 μl) were cycled on a GStorm thermal cycler (98 °C 30 s, 35 cycles of (98 °C 10 s, 60 °C 30 s, 72 °C 15 s), 72 °C 10 min, 10 °C ∞) and purified with a PCR purification kit (Qiagen, #28104). *In vitro* transcription was performed with 300 ng purified DNA template for 4 h at 37 °C using the Megascript T7 kit (Ambion, #AM1334), and sgRNA purified by phenol chloroform extraction and isopropanol precipitation. sgRNAs were diluted to 1 μg μl^−1^ in water and stored in aliquots at −80 °C (refs 16, 27).

### Generation of *ban*-MRE^mut^ mutant stocks

A measure of 0.5 μg sgRNA and 10 μg Cas9 mRNA were precipitated with ethanol to purify and concentrate, and resuspended in water at 0.5–1 μg μl^−1^ for injection. Oregon-R *Drosophila melanogaster* embryos were collected for 30 min at 25 °C, and injected through the chorion at the posterior end. A Femtojet Express (Eppendorf) was used with an Injectman NI2 micromanipulator and Femtotip II needles (Eppendorf, #930000043). Embryos were incubated at 25 °C for the remainder of the development. Putative mosaic flies were crossed individually to *yw*; Sco/CyO flies. These flies were removed after 7 days, and analysed for mutant tissue by HRMA (Fig. 2b). Only crosses with detectable mosaic mutations tissue were carried forward for further analysis. One wing was removed from offspring from these crosses and they were analysed for heterozygous mutations by HRMA and sequencing of the PCR product (Fig. 2b,c). During this process, flies were stored individually in microfuge tubes containing fly food with a hole in the lid. Putative mutants identified by HRMA were crossed individually to yw; Sco/CyO, and stable lines were generated from the offspring of these crosses. Homozygous mutants were validated by sequencing. The following *Drosophila* lines were used for analysis of third instar wing imaginal discs: *dpp*-Gal4, *UAS-CD8-EGFP* (Bloomington Stock Center); *ena*-3′UTR-sensor and *UAS-bantam* were a generous gift from Marco Milan^15^. Further information concerning experimental methods for *Drosophila* CRISPR injections are provided at the OxfCRISPR website ( http://www.oxfcrispr.org)^27^.

### HRMA and sequencing

gDNA was extracted from single flies or single wings by homogenizing in 50 μl or 10 μl squishing buffer (10 mM Tris–HCl, pH=8.2, 1 mM EDTA, 25 mM NaCl, 200 μg ml^−1^ proteinase K (NEB, #P8102), and heating to 37 °C for 30 min or 60 min, followed by inactivation at 95 °C for 2 min (ref. 28). Oligonucleotides enaF2 (5′-AATGAAAACAATTCCACAAACGTC ATCC-3′) and enaR (5′-TAAGTTGCTCGAGCTAACTCTGAGTCC-3′) were used to amplify a 238-nt product spanning the sgRNA target site. Hotshot Diamond PCR mastermix (Clent Lifescience, #HS002) was used to perform PCR in 10 μl reactions with 1 μl gDNA, 5 μl Hotshot Diamond mastermix, 200 nM each oligonucleotide and 1 μl LC Green Plus dye (Idaho Technology, #BCHM-ASY-0005). Reaction solutions were cycled on a GStorm thermal cycler (95 °C 5 min, 45 cycles of (95 °C 20 s, 60 °C 30 s, 72 °C 30 s), 95 °C 30 s, 25 °C 30 s, 10 °C ∞). HRMA was performed on a LightScanner (Idaho Technology; 70–98 °C, hold 67 °C). PCR products from HRMA analysis (5 μl) were treated with 2 μl Exo-SAP IT (Affymetrix, #78200) for 37 °C for 15 min, enzymes inactivated at 80 °C for 15 min and sequenced with the enaF2 primer (5′-AATGAAAACAATTCCACAAACGTCATCC-3′).

### Whole-mount immunofluorescence and imaging

*Drosophila* third instar larvae were dissected in PBS and wing imaginal discs were fixed in 4% paraformaldehyde for 20 min, permeabilized in PBS-T (PBS, 0.1% Triton X-100) and blocked in 5% normal goat serum in PBS-T. Discs were then incubated overnight at room temperature with primary antibodies mouse anti-Enabled (1:200, DSHB, #5G2), rabbit anti-GFP (1:1,000, Invitrogen, #A11122) and mouse anti-wingless (1:200, DSHB, #4D4), washed three times 20 min each in PBS-T and incubated for 2 h at room temperature with anti-mouse A568 (1:1,000, Invitrogen, #11031), anti-rabbit A488 (1:1,000, Invitrogen, #11034) and DAPI (1:2,000, Invitrogen, #D1306). Samples were then mounted in Slowfade (Invitrogen, #S36936) and imaged on a Zeiss 780 confocal microscope with a × 25 oil immersion objective and × 0.7 digital zoom. Images were acquired using similar laser power, pinhole size and gain parameters, and adjusted post-acquisition for background intensity and contrast correction. For assessing Ena expression in the *dpp*-GAL4>*UAS-ban* background, a minimum of 30 third instar larval wing imaginal discs were analysed. The results displayed in Fig. 3 were representative of all samples analysed. For investigation of DV boundary integrity, a minimum of 10 wing imaginal discs or adult wings were analysed from homozygous *ban*-MRE^mut^ lines. To confirm the nature of *ban*-MRE^mut^ indels in animals processed for immunofluorescence and imaging, half of each larvae was snap frozen immediately after dissection and subjected to gDNA extraction followed by PCR validation and sequencing.

### sgRNA design and transfection into mammalian and *Drosophila* cells

Target sites of the form N_20_NGG were chosen as close as possible to the predicted MREs in the 3′UTRs of PCMTD1 (5′-GGCAATATATGAGTGCAATA-3′), MAPRE1 (5′-CCTTGGGATCTGCCAGGCTG-3′) and C9orf7 (5′-GTGGCATCTGAGGCCGGGAG-3′) in human cells, and Pck (5′-TCCATAGTGCCTTTAACAAT-3′) and CG13088 (5′-CATTTCTACCTCAATCCGTC-3′) in *Drosophila* cells. Target sequences were cloned into the pX330 vector (Addgene, plasmid #42230)^29^ or pAc-sgRNA-Cas9 vector (Addgene. plasmid #49330). HDR templates were synthesized as ssDNA oligonucleotides (IDT, Ultramer), with ~60 nt homology arms either side of the inserted sequence (Fig. 4a,b). Sequences were designed to delete either the seed sequence or 3′ motif postulated to be important for MRE function, and replace it with a T7 primer-binding site, or simply insert a T3 primer-binding site immediately downstream of the predicted MRE. Target sequences and the sequences of the ssDNA donor oligonucleotides are provided below (underline=PAM; bold=seed or motif site; italics=T3 or T7 barcode):

PCMTD1 target

5′-TTACTTTTGTTGCAATCACTGTTGTTGGGTTGCTGTATATATATTCCGGGCAATATATGA **GTGCAAT**AACAATACAAGATATTGAATAATTTAGCTTTAAAAAATCCCACAAATTTTATG AAATTTT-3′

PCMTD1 ssOligoT7

5′-TTACTTTTGTTGCAATCACTGTTGTTGGGTTGCTGTATATATATTGCGGGCAATATATGA*TAATACGACTCACTATAGGG*AACAATACAAGATATTGAATAATTTAGCTTTAAA AAATCCCACAAATTTTATGAAATTTT-3′

PCMTD1 ssOligoT3

5′-TTACTTTTGTTGCAATCACTGTTGTTGGGTTGCTGTATATATATTGCGGGCAATATATGA **GTGCAAT***AATTAACCCTCACTAAAGGGA*AACAATACAAGATATTGAATAATTTAGCTTTAAAAAATCCCACAAATTTTATGAAATTTT-3′

MAPRE1 target

5′-CTTTCTGGACCTCTGGCAAAGGGAGTGGTCAGTGAAGGCCATCGTTACCTTGGGATCTGC**CAGGCTGGG**GTGTTTTCGGTATCTGCTGTTCACAGCTCTCCA CTGTAATCCGAATACTTTGCCAGTGCA-3′

MAPRE1 ssOligoT7

5′-CTTTCTGGACCTCTGGCAAAGGGAGTGGTCAGTGAAGGCCATCGTTACCTTGGGATCTGC*TAATACGACTCACTATAGGG*GTGTTTTCGGTATCTGCTGTTCACAGCTCTCCACTGTAATCCGAA TACTTTGCCAGTGCA-3′

MAPRE1 ssOligoT3

5′-CTTTCTGGACCTCTGGCAAAGGGAGTGGTCAGTGAAGGCCATCGTTACCTTGGGATCTGC***CAGGCTGGG**AATTAACCCTCACTAAAGGGA*GTGTTTTCGGTATCTGCTGTTCACAGCTCTCCACTGTAATCCGAATACTTTGCCAGTGCA-3′

C9orf7 target

5′-AACTGTTTCCCAGGAACACCTCTCGGGCCCATCTGCGTCTGAGGCTGGGAGTGGCATCTG**AGGCCGGGA**GTGGCATCTGAGGCCAGGAGTGGCAGGCTGGTGGGCTGGGCGTGGGGTT TTCTGGGCCCT-3′

C9orf7 ssOligoT7

5′-AACTGTTTCCCAGGAACACCTCTCGGGCCCATCTGCGTCTGAGGCTGGGAGTGGCATCTG*TAATACGACTCACTATAGGG*GTGGCATCTGAGGCCAGGAGTGGCAGGCTGGTGGGCTGGGCGTG GGGTTTTCTGGGCCCT-3′

C9orf7 ssOligoT3

5′-AACTGTTTCCCAGGAACACCTCTCGGGCCCATCTGCGTCTGAGGCTGGGAGTGGCATCTG**AGGCCGGGA***AATTAACCCTCACTAAAGGGA*GTGGCATCTGAGGCCAG GAGTGGCAGGCTGGTGGGCTGGGCGTGGGGTTT TCTGGGCCCT-3′

PCK target

5′-ATTGGATCTTAGCTTAAGTTTTCAGACGCAATCTCGTGCCTCGAATCTGCGCAATCGACG**CCGTCC**ATAGTGCCTTTAACAATCGACCATATGTATCTATATACACGCCGACTCAGCCGAG ATCAG-3′

PCK ssOligoT7

5′-ATTGGATCTTAGCTTAAGTTTTCAGACGCAATCTCGTGCCTCGAATCTGCGCAATCGACG*TAATACGACTCACTATAGGG*ATAGTGCCTTTAACAATCGACCATATGTATCTATATACA CGCCGACTCAGCCGAGATCAG-3′

PCK ssOligoT3

5′-ATTGGATCTTAGCTTAAGTTTTCAGACGCAATCTCGTGCCTCGAATCTGCGCAAT CGACG**CCGTCC***AATTAACCCTCACTAAAGGGA*ATAGTGCCTTTAACAATCGACCATATGTATCTATATACACGCCGACTCAGCCGAGATCAG-3′

CG13088 target

5′-GAATTCAGTCCAGACTCGTAGTAGTCATTTGAAAAGACTTAAATGACATTTCTACCTCAA** TCCGTCC**GGTATACGAATATATATGTAGATGGAGATCCAA ATGATATATCCTGAGTAAAATGTTGTA-3′

CG13088 ssOligoT7

5′-GAATTCAGTCCAGACTCGTAGTAGTCATTTGAAAAGACTTAAATGACATTTCTACCTCAA* TAATACGACTCACTATAGGG*GGTATACGAATATATATGTAGATGGAGATCCAAATGATATATCCTGAG TAAAATGTTGTA-3′

CG13088 ssOligoT3

5′-GAATTCAGTCCAGACTCGTAGTAGTCATTTGAAAAGACTTAAATGACATTTCTACCTCAA **TCCGTCC***AATTAACCCTCACTAAAGGGA*GGTATACGAATATATATGTAGATGGAGATCCAAATGATATATCCTGAGTAAAATGTTGTA-3′

HEK293T cells were cultured at 37 °C and 5% CO_2_ in DMEM with 25 mM glucose (Gibco, #41966-029), 10% fetal bovine serum (Gibco, #10270) and 1% penicillin-streptomycin (Gibco, #15140-122). S2R+ cells were cultured at 25 °C in Schneider’s *Drosophila* medium (Sigma, #S9895), 10% fetal bovine serum (Gibco, #10270) and 1% penicillin-streptomycin (Gibco, #15140-122). Cas9/sgRNA expression vectors (1 μg) and homology oligonucleotides (0.5 μg each T3/T7 oligo) were co-transfected into HEK293T cells in a six-well dish using polyethylenimine (Sigma-Aldrich) as previously described^30^. Transfection of S2R+ cells was performed similarly but with Fugene HD (Promega) using a 1:3 ratio (μg DNA:μl Fugene) following the manufacturer’s recommendations. Cells were collected 72 h post transfection and washed three times in PBS, after which one-third of the cells were used for gDNA extraction, and two-thirds used for RNA extraction. Site-specific integration of the T3- and T7-containing donor oligos was verified by Sanger sequencing. Average integration efficiencies of T3 and T7 oligos were 0.22% (s.e.m.=0.044%) and 0.33% (s.e.m.=0.093%), respectively.

### gDNA and RNA extraction

gDNA was extracted from zebrafish embryos by addition of 300 μl lysis buffer (10 mM Tris-HCl, pH=7.5, 10 mM NaCl, 10 mM EDTA, 0.5% sarcosyl) containing 200 μg ml^−1^ of proteinase K (Invitrogen, #25530-049), and incubation at 55 °C for a minimum of 2 h. Proteins were removed by phenol/chloroform extraction, and DNA precipitated with 2.5 volumes ethanol. DNA was quantified with a Nanodrop spectrophotometer and diluted to a concentration of 100 ng μl^−1^ before analysis. RNA was extracted using a miRNeasy mini kit (Qiagen, #217004) including the additional DNAse treatment step. cDNA was synthesized from 1 μg RNA with the Quantitect Reverse Transcription kit (Qiagen, #205311) and diluted twofold before analysis.

### T7/T3 expression analysis

Quantitative PCR was performed with gene-specific forward primers (PCMTD: 5′-GCAGTTGTTTGCATTTCCTCTATG-3′, MAPRE1: 5′-TGCTGCTTAGAGTTGGAAGTGC-3′, C9orf7: 5′-GCTTCTGGAGCGCAGGTACTG-3′, Pck: 5′-CCTGCAGGGCTACATATAACAGC-3′, CG13088: 5′-GAGTCCGCGATTTGTGGAAA-3′), and either T3as (5′-TCCCTTTAGTGAGGGTTAATT-3′) or T7as (5′-CCTATAGTGAGTCGTATTA-3′) reverse primers. Amplification was performed with SybrGreen JumpStart Taq Ready Mix (Sigma, #S4438) and detected on an ABI 7500 Fast thermal cycler. Results are shown as a ratio of the signal with the T7 primer to that with the T3 primer for gDNA and cDNA for three independent biological replicates, each analysed in technical triplicate. The final signal ratio was obtained by calculating the difference in Ct between T7 and T3 primers (ΔCt) during the exponential amplification phase, and subsequently transforming the resulting values using 2^−ΔCt^.

### Genome-wide CRISPR target site prediction at MREs

MREs for conserved miRNAs with a ‘good mirSVR score’ were predicted using the miRanda algorithm (http:// www.microrna.org, August 2010 Release) for four species, *Drosophila*, human, mouse and rat. A custom perl script was used to identify potential CRISPR target sites within a 200-bp window around each of the MREs from the appropriate genomic sequences (dm3, hg19, mm9, rn4, respectively). Potential off-targets for each of the CRISPR target sites were identified using the BatMis algorithm^31^ allowing up to four mismatches, and a subsequent step to look for the presence of an appropriately positioned PAM (NGG or NAG) site. CRISPR target sites that had off-targets with fewer than two mismatches were rejected. The nearest CRISPR target sites to each MRE and any associated off-target locations were recorded.

## Author contributions

T.A.F. and A.R.B. conceived and designed the experiments. T.A.F., A.R.B., G.A., L.W., C.T., T.R., N.S. and T.S.-S. performed the experiments. T.A.F., A.R.B. and T.S.-S. analysed the data and interpreted the results. T.A.F., S.McG. and S.T. designed the miR-CRISPR algorithm. S.McG. and S.T. performed the bioinformatics analysis and generated the miR-CRISPR online resource. P.A.E. coded the miR-CRISPR user interface. T.A.F. and A.R.B. wrote the manuscript and generated the figures and C.P.P., T.S-S. and J.-L.L. edited them.

## Additional information

**How to cite this article:** Bassett, A. R. *et al.* Understanding functional miRNA–target interactions *in vivo* by site-specific genome engineering. *Nat. Commun.* 5:4640 doi: 10.1038/ncomms5640 (2014).

## Supplementary Material

Supplementary Information Supplementary Figure 1 and Supplementary Table 1
