# Supplementary material for: Understanding functional miRNA–target interactions in vivo by site-specific genome engineering
Source: Nat Commun. 2014 Aug 19;5:4640. doi: 10.1038/ncomms5640 (PMC4143950; doi:10.1038/ncomms5640)
Supplement: Supplementary Information — Supplementary Figure 1 and Supplementary Table 1 [file ncomms5640-s1.pdf]

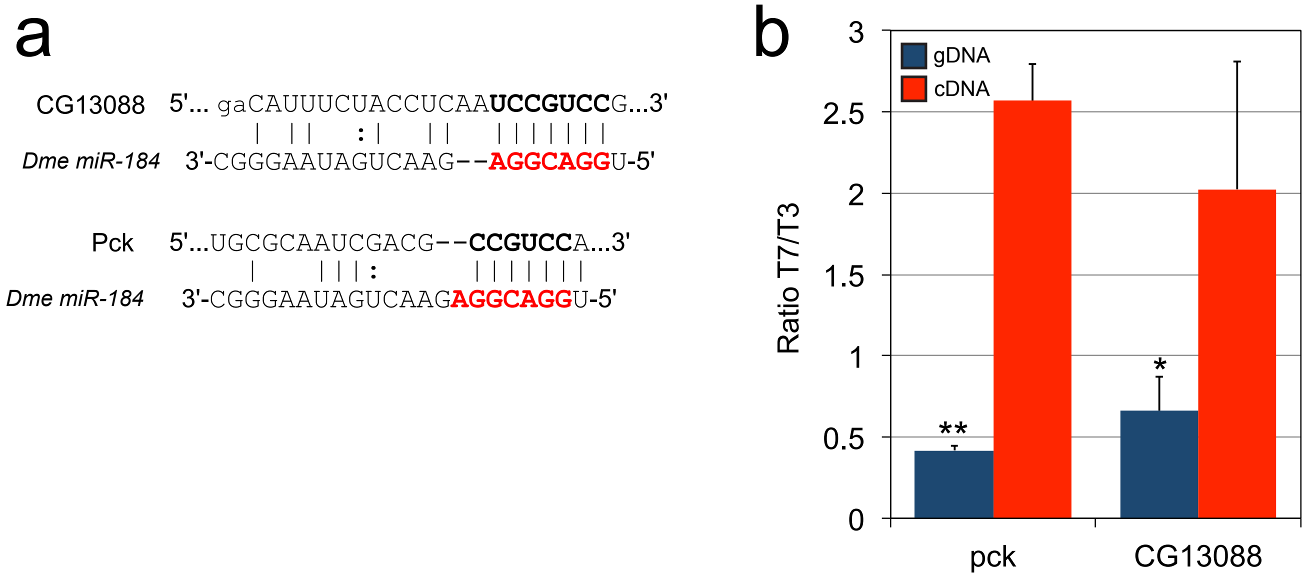

Supplementary Figure 1: Analysis of MRE activity in *Drosophila* S2R+ cells in culture. **(a)** Two predicted targets of miR-184, PCK and CG13088 were tested in this system. Sequences deleted by HDR are shown in bold; the miR-184 seed sequence is in red. **(b)** The ratio of T7 signal to T3 signal is displayed on the y-axis, and results from gDNA are shown in blue and cDNA in red. Both transcripts appear to be upregulated upon MRE deletion in this cell type ($p<0.05$, one-tailed T-test, n=3).

	WT		T7 MRE^{mut}		T3 MRE^{WT}	
	microRNA	Seed	microRNA	Seed	microRNA	Seed
PCMTD1	hsa-miR-92b	8mer	hsa-miR-513a-3p	8mer	hsa-miR-92b	8mer
	hsa-miR-92a	8mer	hsa-miR-1290	8mer	hsa-miR-92a	8mer
	hsa-miR-513a-3p	8mer	hsa-miR-944	7mer	hsa-miR-513a-3p	8mer
	hsa-miR-367	8mer	hsa-miR-643	7mer	hsa-miR-367	8mer
	hsa-miR-25	8mer	hsa-miR-921	7mer	hsa-miR-25	8mer
	hsa-miR-1290	8mer			hsa-miR-1290	8mer
	hsa-miR-944	7mer			hsa-miR-944	7mer
	hsa-miR-643	7mer			hsa-miR-643	7mer
	hsa-miR-363	7mer			hsa-miR-363	7mer
	hsa-miR-32	7mer			hsa-miR-32	7mer
					hsa-miR-944	8mer
					hsa-miR-921	8mer
					hsa-miR-211	7mer
					hsa-miR-204	7mer
C9orf7	hsa-miR-596	8mer	hsa-miR-596	8mer	hsa-miR-596	8mer
	hsa-miR-1226	8mer	hsa-miR-1226	8mer	hsa-miR-1226	8mer
	hsa-miR-483-3p	7mer	hsa-miR-483-3p	7mer	hsa-miR-483-3p	7mer
			hsa-miR-921	7mer	hsa-miR-921	8mer
					hsa-miR-211	7mer
					hsa-miR-204	7mer
MAPRE1	hsa-miR-548l	8mer	hsa-miR-548l	8mer	hsa-miR-548l	8mer
	hsa-miR-211	8mer	hsa-miR-211	8mer	hsa-miR-211	8mer
	hsa-miR-204	8mer	hsa-miR-204	8mer	hsa-miR-204	8mer
	hsa-miR-922	7mer	hsa-miR-922	7mer	hsa-miR-922	7mer
	hsa-miR-570	7mer	hsa-miR-570	7mer	hsa-miR-570	7mer
			hsa-miR-921	7mer	hsa-miR-921	8mer
					hsa-miR-211	7mer
					hsa-miR-204	7mer

Supplementary Table 1: Comparative miRNA target predictions before and after T7 or T3 barcode integration. MRE prediction was performed using the PITA algorithm for WT sequences and insertions of the T7 MRE^{mut} and T3 MRE^{WT} HDR oligonucleotides. For T7 and T3 integrant sequences, MREs added to the WT sequence are highlighted in grey. The miR-944 MRE differentially generated by the T3 MRE^{WT} in PCMDT1 is unlikely to be functional since miR-944 is not expressed to any degree in HEK-293 cells.
